# Evaluating Imaging Techniques for Diagnosing and Drainage Guidance of Psoas Muscle Abscess: A Systematic Review

**DOI:** 10.3390/jcm13113199

**Published:** 2024-05-29

**Authors:** Murtadha Qais Al-Khafaji, Mohammad Walid Al-Smadi, Mustafa Qais Al-Khafaji, Siran Aslan, Yousif Qais Al-Khafaji, Panna Bagossy-Blás, Mohammad Hakem Al Nasser, Bálint László Horváth, Árpád Viola

**Affiliations:** 1 Faculty of Medicine, University of Debrecen, 4032 Debrecen, Hungary; murtadha@mailbox.unideb.hu (M.Q.A.-K.); mustafaqais@mailbox.unideb.hu (M.Q.A.-K.); yousifqaismuhsunalkhafaji@mailbox.unideb.hu (Y.Q.A.-K.);; 2 Department of Neurosurgery and Neurotraumatology, Dr. Manninger Jenő National Traumatology Institute, 1081 Budapest, Hungary; smadi996@hotmail.co.uk (M.W.A.-S.); drsiran.5@gmail.com (S.A.); b.blas.panna@gmail.com (P.B.-B.); 3 Department of Neurotraumatology, Semmelweis University, 1081 Budapest, Hungary; 4 Doctoral School of Clinical Medicine, Semmelweis University, 1083 Budapest, Hungary; 5 Department of Traumatology, Dr. Manninger Jenő National Traumatology Institute, 1081 Budapest, Hungary; thebe88@gmail.com

**Keywords:** psoas muscle abscess, image-guided percutaneous drainage, computed tomography (CT), magnetic resonance imaging (MRI), fusion technique, ultrasound (US), psoas muscle infection, percutaneous intervention, surgical drainage, psoas muscles

## Abstract

**Background:** Psoas muscle abscess (PMA) is an uncommon yet severe condition characterized by diagnostic and therapeutic challenges due to its varied etiology and nonspecific symptoms. This study aimed to evaluate the effectiveness and accuracy of various imaging techniques used in the image-guided percutaneous drainage (PD) of PMA. **Methods:** A systematic review was conducted following the PRISMA guidelines. We searched PubMed, Google Scholar, and Science Direct for studies published in English from 1998 onwards that reported on the use of PD in treating PMA, detailing outcomes and complications. Imaging modalities guiding PD were also examined. **Results:** We identified 1570 articles, selecting 39 for full review. Of these, 23 met the inclusion criteria; 19 were excluded due to unspecified PMA, absence of imaging guidance for PD, or inconclusive results. Eleven studies utilized computed tomography (CT) for PD, with six also using magnetic resonance imaging (MRI). Ten studies implemented ultrasound (US)-guided PD; variations in diagnostic imaging included combinations of US, CT, and MRI. A mixed approach using both CT and US was reported in two articles. Most studies using CT-guided PD showed complete success, while outcomes varied among those using US-guided PD. No studies employed MRI-guided PD. **Conclusions:** This review supports a multimodal approach for psoas abscess management, using MRI for diagnosis and CT for drainage guidance. We advocate for Cone Beam CT (CBCT)-MRI fusion techniques with navigation systems to enhance treatment precision and outcomes, particularly in complex cases with challenging abscess characteristics.

## 1. Introduction

Psoas muscle abscess (PMA) is an uncommon yet severe infection characterized by pus accumulation within the iliopsoas muscle, which can lead to significantly high morbidity and mortality rates. Despite its rarity, with an incidence reported as four in one million annually in the UK and a mortality rate reaching up to 19% [1,2,3,4], males are more commonly affected than females, with a ratio of 1.62:1 [5,6]. PMA poses diagnostic and management challenges, mainly due to its varied etiology and vague clinical presentation with nonspecific symptoms, such as fever, limited hip movement, and back pain, which are present in only 30% of cases [4,7]. PMA manifests primarily in two forms [8,9]: primary, resulting from hematogenous or lymphatic spread [8,10], more prevalent in Asia and Africa, and secondary, associated with adjacent infections or conditions like Crohn’s disease and pyogenic spondylodiscitis, more common in Europe [11,12,13,14].

The diagnosis of PMA relies heavily on imaging techniques, with computed tomography (CT) being the gold standard due to its high accuracy [3,15,16]. However, other methods like ultrasound (US) and magnetic resonance imaging (MRI) offer the potential for early detection without ionizing radiation exposure [3,11,17,18,19]. Despite advancements, the management of PMA remains a challenge, primarily focusing on early and appropriate antibiotic administration coupled with efficient abscess drainage, either percutaneously or surgically, based on the abscess characteristics and the patient’s overall condition [3,8,9].

Following the indications put forth by [20], open surgical drainage (OS) is recommended in cases where percutaneous drainage (PD) proves ineffective, when PD poses relative contraindications specifically when the abdominal condition presents with another disease that necessitates surgical intervention, such as Crohn’s disease, or when dealing with multiloculated abscesses [3]. It has been noted that open surgical drainage tends to yield superior outcomes concerning achieving complete drainage compared to PD [5]. PD is more effective and less invasive than open surgical drainage. It is the method of choice for drainage and the primary initial treatment [15,21,22,23,24]. PD is often image-guided, where imaging modalities such as US, CT, and MRI guidance can be used [5,21]. The use of these imaging methods in guiding PD has been highly recommended lately. This study aims to evaluate the accuracy and effectiveness of using different imaging methods in the PD of PMA.

This study aimed to review and analyze the different imaging modalities in guiding the PD of PMA and the related diagnostic methods by evaluating their accuracy and effectiveness. The focus on imaging-guided PD reflects a critical step toward optimizing PMA management. However, we believe there is still space for improvement in guiding the PD process using fusion techniques (CT and MRI), especially since it has proven its efficacy in different percutaneous interventions, such as diagnosing and ablating different spinal column tumors or their management [25,26].

## 2. Materials and Methods

**Figure 1:**
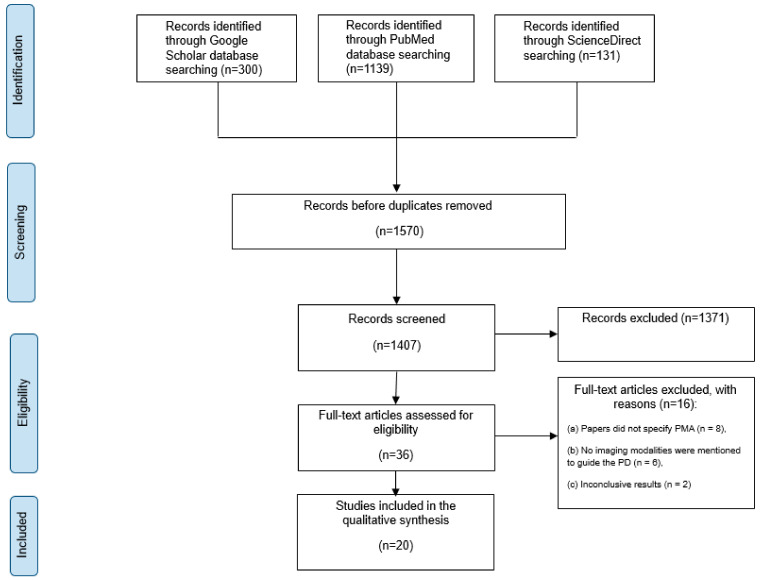
An overview of the study selection based on PRISMA.

This systematic review was carried out by the Preferred Reporting Items for Systematic Reviews and Meta-Analyses (PRISMA) guidelines (Figure 1). A comprehensive electronic search of PubMed, Google Scholar, and Science Direct databases was conducted for studies published between 1998 and October 2023. The search strategy was developed following the traditional PICO method of searching electronic databases. The systematic review followed a priori eligibility criteria, and the protocol was registered in the PROSPERO International Prospective Register of Systematic Reviews (CRD42024511906).

Using the Medical Subject Heading (MeSH), a combination of the following keywords was used: (“Psoas muscle abscess”, or “Psoas Muscles”, or “Psoas muscle infection”, or “Iliopsoas abscess”) and (“Percutaneous drainage” or “Percutaneous Intervention” or “Surgical treatment” or “Surgical intervention” or “Surgical drainage”) and (“Ultrasound” or “CBCT” or “X-ray” or “X-ray computed” or “CT” or “Computed Tomography” or “Tomography” or “MRI” or “Magnetic Resonance Imaging” or “Imaging modalities” or “Imaging diagnostics” or “Diagnostic Imaging” or “Radiological”). 

### 2.1. Eligibility Criteria

PMA studies for patients with only PD published in English were included. Studies were required to report patient outcomes, case series, or original articles, including randomized controlled trials (RCTs), prospective cohort studies, retrospective cohort studies, and non-randomized non-comparative studies. Those studies that included less than 5 patients undergoing percutaneous drainage were excluded. The primary outcome of interest is the imaging modality used to diagnose and treat the abscess with PD. Also, we looked at the outcomes, which include post-operation improvement and adverse events related to the PD of PMA. Studies that did not report their outcomes were excluded.

The exclusion criteria included studies that investigated surgical interventions other than PD, studies that did not mention the use of imaging modalities that guide PD in treating PMA, improper methods (reported a meta-analysis/systematic review, economic analysis, animal study, cadaver study, narrative review, or editorial), and studies that reported no outcomes of interest.

### 2.2. Selection of Articles and Data Extraction

Four reviewers independently assessed the titles and abstracts obtained from the search strategy using the Massachusetts, USA-based software Rayyan (http://rayyan.qcri.org, accessed on 7 October 2023) following the study’s eligibility requirements. Following that, all authors evaluated the full texts of the selected abstracts individually to determine final eligibility. When there was disagreement, the reviewers worked together to reach a consensus.

This process ensured that only relevant and appropriate studies were included in the final research analysis, improving the accuracy and reliability of the study selection. The title, study design, country, age range, mean age in years, gender, side of abscess, number of PMA, number of PD, number of patients treated for percutaneous psoas abscess drainage, image modality, size of abscess in CT, size of abscess in MRI, complications, primary or secondary, primary (surrounding tissue or dissemination), etiology, cause of infection/microorganism, presence of immunodeficiency, duration of operation, conservative treatment if applicable, way of treatment (surgical or percutaneous), equipment used, duration of catheter effectiveness (length of hospital stay), outcome, efficiency, efficacy, accuracy, recurrence, indicate who performed the procedure, where it took place (hospital, surgery clinic), follow-up period, and overall conclusion and clinical recommendation from each study.

The success of the PD is determined by the absence of reported recurrence and the adequacy of the drainage performed. Additionally, the lack of complications is a key indicator of success; however, as most articles did not report on complications, we primarily considered the absence of recurrence as an indication that the drainage volume was sufficient.

### 2.3. Evaluation of the Statistical Data

Despite our attempt at a rudimentary descriptive statistical analysis, the heterogeneity of the included articles and the absence of data in a format conducive to meta-analysis made it unfeasible to conduct a meta-analysis. 

## 3. Results

### 3.1. Search Results

Initially, 1570 papers were collected from the three databases mentioned above. A total of 1371 papers were removed after thorough abstract screening and duplicate removal. Among the remaining 39 papers, 19 were excluded based on the inclusion and exclusion criteria. This exclusion was due to 11 studies that did not specifically focus on patients with PMA. Six studies did not mention the guided imaging modality, and two had inconclusive results (Figure 1).

### 3.2. Demographic and Clinical Data of All Patients

**Figure 2:**
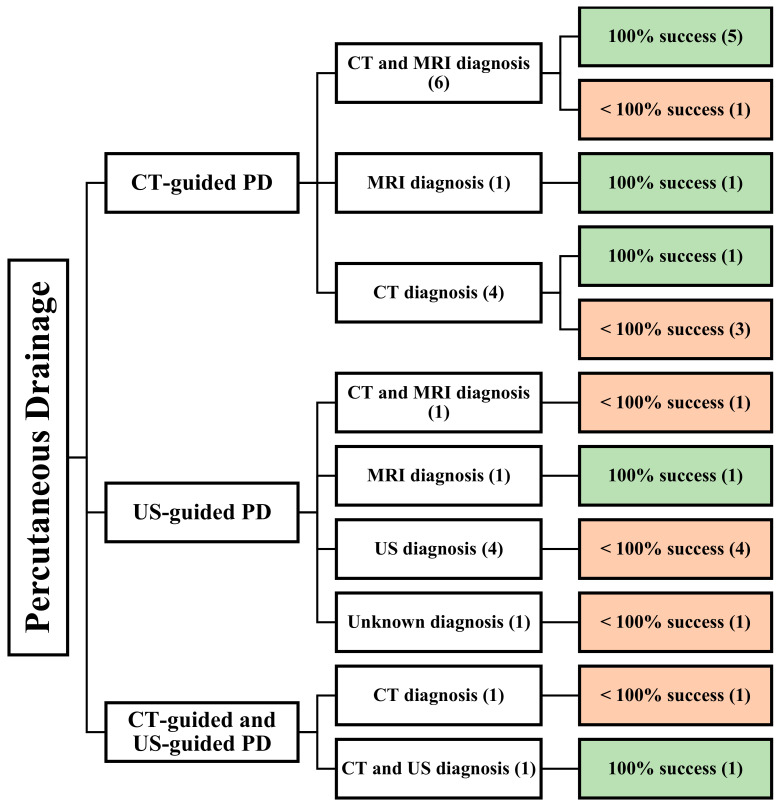
An overview of all patients undergoing image-guided PD. () the number of articles.

A total of 20 articles met the eligibility criteria and were reviewed. They comprise 19 retrospective studies and 1 prospective study, of which all were cohort studies. A total of 338 patients were included in those studies. The mean age of all the patients was 36 years. Only 190 patients had their gender described, and 103 males and 87 females were evaluated at a mean follow-up of 23.5 months. Twelve studies used CT-guided PD [21,22,27,28,29,30,31,32,33,34,35], seven studies used US-guided PD [12,36,37,38,39,40,41], and two studies used both CT-guided and US-guided PD [42,43] (Figure 2).

### 3.3. Demographic and Clinical Data of CT-Guided PD

**Figure 3:**
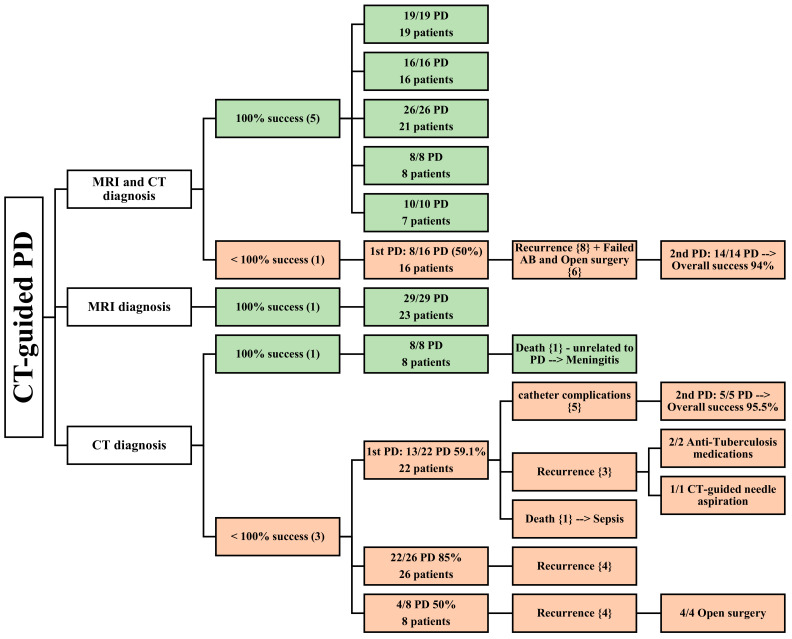
An overview of patients undergoing CT-guided PD. () the number of articles; {}the number of patients.

A review of 20 articles found that 12 articles focused on CT-guided PD [21,22,27,28,29,30,31,32,33,34,35]. These studies were all retrospective, and a total of 181 patients were included in the analysis. Among these articles, six utilized both CT and MRI for diagnosing PMA [22,27,29,30,32,35], while four relied solely on CT [21,28,31,34], and one utilized MRI [33] (Figure 3). 

#### 3.3.1. CT and MRI as Diagnostic Methods

Five articles reported a 100% success rate for CT-guided PD [22,27,30,32,35]. CT and MRI were used to diagnose 71 patients in total, who underwent successful CT-guided PD. Three of these papers [22,30,35] reported a mean follow-up time of 18.9 months. Four papers [22,30,32,35] described 52 patients, 18 of whom had bilateral PMA; the mean patient age was 48.975 years, and 32 male and 20 female patients were included in these studies. In one article [27], the abscesses were all located in the iliopsoas muscle, while in two other articles [22,32], they were located in both the iliopsoas and paravertebral muscles. One article had specifically outlined the location of the abscesses according to the spine level involved [35]. Three studies described the condition’s etiology [22,30,35], and the two most common causes were spinal tuberculosis and spondylodiscitis. One article stated the comorbidities in six patients undergoing PD who had immunodeficiency, including renal failure, HIV, and intravenous drug use, while the other four studies did not mention any [30]. 

The size of the abscesses was mentioned in two studies [30,32], with a mean diameter of 5.7 cm. The volumes of the abscesses drained were mentioned in two articles, which were more than 100 mL [35] and 321 mL [22], while the other three articles did not specify [27,30,32]. In contrast to earlier findings, however, the last article reported incomplete success of CT-guided PD [29]. This study reported a 50% success rate in the first PD (8 out of 16 cases), with eight recurrences, while the second PD achieved a success rate of 100% (14 cases). US was also used as a diagnostic imaging modality. A total of 23 patients were diagnosed using both CT and MRI. The mean patient age was 62, and the male-to-female ratio was roughly similar, with slightly more males than females (12 males and 11 females). 

The location, comorbidities, drained abscess volume, and follow-up period were not assessed in this study. The most common etiology was spondylodiscitis, followed by gastrointestinal (GI) infections and postoperative septic complications (Table 1).

#### 3.3.2. MRI as a Diagnostic Method

One paper relied solely on MRI for diagnostic purposes, and CT-guided PD demonstrated complete success in all 23 patients who were evaluated, with a mean follow-up period of 27.7 months [33]. Among these patients, six had bilateral PMA, while the rest had either right (6) or left (11) PMAs. The study population consisted of 23 patients (13 males and 10 females), with a mean age of 69 years. The etiology identified in this study was pyogenic spondylitis, and all PMAs were secondary abscesses with a mean diameter of 4.8 cm. Seven of these patients had diabetes, while three patients had renal failure (Table 1). The average drained abscess volume was not stated in this article. 

#### 3.3.3. CT as a Diagnostic Method

Four studies utilized CT as a diagnostic modality [21,28,31,34]. One of these studies reported a complete success rate for PD in eight patients, with only one patient experiencing a bilateral abscess [31]. One patient had their PMA located at the lumbar level, while the other abscesses were located at both the lumbar and pelvic levels. The average abscess size was 9 cm. However, this study failed to evaluate the patients’ age, male-to-female ratio, etiology, drained abscess volume, comorbidities, and follow-up period. 

In contrast, the remaining three studies, which included 57 patients, reported abscess recurrence [21,28,34], death [21], and other catheter complications (dislocation or obstruction) [21], resulting in lower success rates for CT-guided PD. One article reported a mean patient age of 36 years, involving 10 male and 11 female patients, with 1 abscess being a bilateral abscess, 2 located in the iliac muscle, 14 located in the psoas muscle, and 6 located in the iliopsoas muscle [21]. 

*Mycobacterium tuberculosis* and *Staphylococcus aureus* were the main causative agents, and the mean follow-up time was 49.6 months. One article mentioned an average of 890 mL of drained abscess from its patients, including two patients with diabetes and systemic lupus erythematosus (SLE). In the other two studies, age, the male-to-female ratio, location, volume of drained abscess, comorbidities, follow-up period, and etiology could not be determined because not all patients underwent PD [28,34]. The average size of the PMAs mentioned in the two studies was 9 cm and 6 cm [21,34] (Table 1).

### 3.4. Demographic and Clinical Data of US-Guided PD

**Figure 4:**
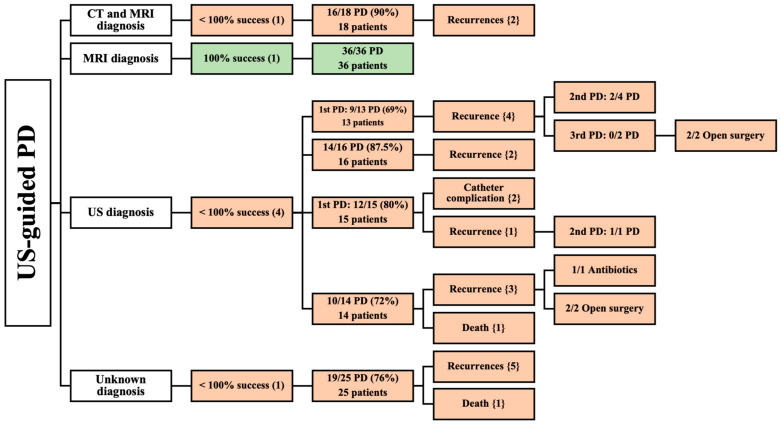
An overview of patients undergoing US-guided PD. () the number of articles; {} the number of patients.

Seven studies reported using US-guided PD in 137 patients [12,36,37,38,39,40,41]. Among these, one study was conducted prospectively [37], and six studies were conducted retrospectively [12,36,38,39,40,41]. Concerning the diagnostic modality, one study utilized both CT and MRI [36], four studies used US [12,38,39,40], one study used MRI [37], and one study did not specify the method of diagnosis [41]. Notably, two studies reported on pediatric patients [39,40] (Figure 4).

#### 3.4.1. CT and MRI as Diagnostic Methods

One articles reported the results of 18 patients diagnosed by CT and MRI [36]. This article reported a high success rate (90%) of US-guided PD in 18 patients. Recurrence occurred in two patients. There was no mention of age, the male-to-female ratio, location, size of the abscess, volume of the drained abscess, etiology, comorbidities, or follow-up period (Table 2).

#### 3.4.2. MRI as a Diagnostic Method

Additionally, MRI was the sole diagnostic imaging modality in one prospective study [37] that reported complete success of US-guided PD on 36 patients. The patients were followed-up for an average of 13 months; their mean age was 42.5 years. The study revealed a slight preponderance of male patients (20 males and 16 females). Spinal tuberculosis was the primary cause of PMA (Table 2). This study did not assess location, the size of the abscess, volume of the drained abscess, or comorbidities. 

#### 3.4.3. US as a Diagnostic Method

A total of 58 patients were included in four studies that utilized US for diagnosis [12,38,39,40]. None of these studies achieved complete success of PD, due to them having multiple recurrences [12,38,39,40], catheter complications [38], and death [39]. Among these, one study [39] that assessed 14 pediatric patients reported a mean age of 6 years for all its patients who underwent US-guided PD. This study included six male and eight female patients, with two patients having bilateral iliopsoas muscle abscesses. Most abscesses (80%) were primary, while the remaining (20%) were secondary, resulting from focal bacterial nephritis and ipsilateral septic arthritis. Two articles stated the volumes of the drained abscesses, which were in the range of 10–100 mL [39] and 20–300 mL [40]. However, the study did not evaluate follow-up time [39]. In contrast, the other three studies [12,38,40] involved 44 patients. They did not specify mean age, gender, location, etiology, comorbidities, or follow-up of patients undergoing US-guided PD. At the same time, two of these articles [12,38] did not state the volume of the drained abscess (Table 2).

#### 3.4.4. Unknown Imaging Modality as a Diagnostic Method

One prospective study paper did not provide information on the diagnostic modality used and included 25 patients with PMA who were treated with US-guided PD, achieving a success rate of 76% [41]. The abscesses of five of these patients recurred, and one patient was deceased. Mean age, gender, location, etiology, size of abscess, volume of drained abscess, comorbidities, nor follow-up period were reported in this study (Table 2).

### 3.5. Demographic and Clinical Data of CT- and US-Guided PD

**Figure 5:**
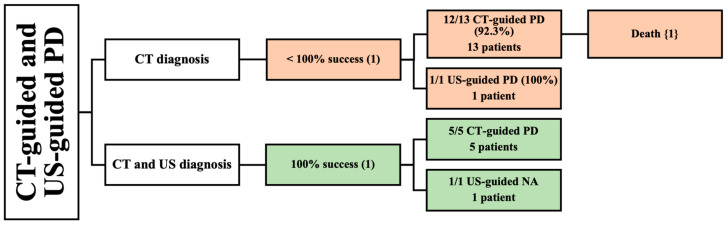
An overview of patients undergoing both CT-guided and US-guided PD. () the number of articles; {} the number of patients.

Two retrospective studies used CT-guided and US-guided PD in 20 patients [42,43]. One study used CT for diagnosis [42], while the other used both CT and US [43] (Figure 5).

#### 3.5.1. CT as a Diagnostic Method

One study described the CT diagnosis of PMA in 14 patients with a mean age of 70 [42]. A total of 5 of the 14 patients had bilateral PMA. The study included seven males and seven females. One patient underwent a successful US-guided PD. On the other hand, 13 patients underwent CT-guided PD, and most of them reported complete success; however, 1 patient died due to an inoperable perforation of diverticulitis, resulting in a lower success rate of 92.3%. The etiologies reported in this study were spondylodiscitis, GI perforation, urinary tract infection (UTI) spreading, and postoperative abscess. Many patient comorbidities were described in this article, including diabetes, thyroid cancer, ovarian cancer, hypertension, urolithiasis, dyslipidemia, liver cirrhosis, and spinal cord injury. Follow-up period was not reported in this study (Table 3). The male-to-female ratio, location size of abscess, and volume of drained abscess were not assessed.

#### 3.5.2. CT and US as Diagnostic Methods

Lastly, one study reported the use of both CT and US to diagnose PMA [43]. The study included six patients with a mean age of 43 years. Two PMAs occurred in male subjects and four PMAs occurred in females. Only one patient had a bilateral abscess. All patients reported complete success with CT-guided PD and US-guided needle aspiration. Tuberculosis was the primary etiology of PMA in this study. One patient had multiple comorbidities, such as diabetes, severe cardiac disease, and arteriopathy. The volume of drained abscess and follow-up period were not mentioned in this study (Table 3).

## 4. Discussion

This systematic review aimed to analyze the recent literature and evaluate the effectiveness of using different imaging modalities to diagnose PMA and guide its PD.

The mean age of PMA patients who underwent image-guided PD was 36 years. As reported in a review by Lai et al., the mean age was 52.7 years in 555 patients with PMA [6], which is quite similar to our findings. Two studies reported a higher tendency for PMA to occur in older individuals [44,45].

As reported in earlier studies [16,46,47,48], PMA is more preponderant in males than in females. We also noticed a higher incidence of male patients (103 cases, 54%) than females (87 cases, 46%).

Bilateral PMA was previously considered a rare entity (3%) [49]. However, the recent literature suggests a higher incidence of 25% [44] and 16% [19]. Suzuki et al. hypothesized that the recent technological advances in diagnostic imaging contributed to the increase in diagnosing bilateral abscesses [42]. Our systematic review included 34 bilateral PMA patients out of 338 patients (10%).

PMA is a rare but severe condition with variable clinical presentation and a reported annual mortality rate of up to 19% [8]. It can be either primary or secondary, caused by a known etiology. *Staphylococcus aureus* is common in primary cases, while streptococci and *E. coli* are prevalent in secondary cases [50]. Comorbidities like diabetes, AIDS, and renal failure are common associated risk factors. Due to their insidious nature, PMAs are usually diagnosed late, thus leading to complications such as septic arthritis, osteomyelitis, and death. Therefore, imaging modalities, including US, CT, and MRI, are crucial for early diagnosis [51]. Treatment involves early antibiotics and drainage, and while open surgical drainage is recommended in specific cases, PD is considered to be the preferred initial method of the management of PMAs [21,29,43].

Historically, the treatment of PMA involved a surgical procedure via retroperitoneal access to remove the abscess and necrotic tissue, supplemented by appropriate antibiotic therapy [52]. However, advancements in imaging technologies and increased expertise in minimally invasive techniques among radiologists have made this approach the preferred method due to its association with lower morbidity and mortality and reduced hospital stays. Nevertheless, this method has limitations, including its suitability for patients with severe sepsis who need quicker resolution of the abscess and those with thick abscess collections, though the latter can often be managed with fibrinolytic agents [53]. Additionally, for PMAs that are secondary to underlying abdominal conditions such as diverticulitis, a surgical approach is favored to address the root cause effectively [9].

When comparing CT-guided and US-guided PD, in which both techniques employ CT and MRI for the diagnosis of PMA, our findings indicate a notably higher efficacy of CT-guided PD (83.3%) in the treatment of the abscess compared to its US-guided counterpart (33.3%). Specifically, across the reviewed studies, CT-guided PD demonstrated a complete success rate in 5 out of 6 articles, including 94 patients, and 71 of them had a successful PD. In contrast, US-guided PD did not achieve complete success in one article, which involved 18 patients, of which only 16 had a successful PD. Although the sample size for the US-guided PD is small compared to that of CT-guided PD, the ratio of successful PD among the CT-guided group is still higher, demonstrating better outcomes. Several studies recommend using US for guiding PD [12,39]. However, our review demonstrated that CT-guided PD is more efficacious. CT-guided PD is a safe and effective first-line procedure that allows for the minimally invasive treatment of abdominopelvic abscesses, particularly in deeper or more posterior parts that are difficult to reach by US-guided methods [29,32,54]. Some advantages of CT-guided PD over US-guided PD include better vision in larger patients, vision not being obscured by gas, and the ability to safely access areas poorly visualized in US [55].

MRI is a valuable diagnostic tool for psoas abscesses, providing helpful information for early diagnosis and precise localization [48,56,57]. Our study assessed the CT-guided and US-guided PD of PMAs that MRI exclusively diagnosed. Our comparison revealed equivalent success rates between the two techniques. In this context, CT-guided and US-guided PD demonstrated complete success in all patients involved (23 and 36, respectively). The analysis encompassed two studies, wherein one employed CT-guided PD while the other utilized US-guided PD. Early and accurate diagnosis of PMA can be achieved by MRI, improving the clinical outcome [58]. 

CT plays a significant role in diagnosing PMA and is considered the gold standard [16,28]. Six studies in our review utilized CT as a diagnostic tool and employed CT- or US-guided PD. When comparing these subgroups, one study reported complete success with the CT-guided technique in eight patients, while another study that employed both guided techniques reported complete success in one patient treated with US guidance and incomplete success among thirteen patients treated with CT guidance due to the death of a patient from perforated diverticulitis [42]. 

In evaluating psoas abscesses, the selection between MRI and CT scans is shaped by medical factors and the uneven global distribution of technological resources. While MRI provides enhanced soft tissue visualization, it is less accessible than CT scans, especially in areas with limited resources due to its higher costs and more complex operational demands. This uneven distribution impacts the availability of diagnostic methods and requires an adaptable approach that considers the specific resources of each medical setting. Therefore, our review highlights the benefits of each imaging modality based on clinical effectiveness. However, it is essential to acknowledge how equipment availability can significantly dictate the choice of diagnostic method.

Far more studies reported incomplete success, among which were three CT-guided studies including 56 patients, with only 39 demonstrating complete success, and one US-guided study on 1 patient, which showed multiple recurrences even with subsequent open surgical drainage [51]. These results show that CT alone as a diagnostic tool is less efficient compared to when it is used alongside MRI, which shows a significantly higher success of image-guided PD.

The limitations of the existing literature on the efficacy of the CT-guided PD of psoas muscle abscesses should be recognized. Notably, the absence of detailed information on the anatomical localization and size of abscesses in many studies may influence the generalizability of our findings. It is essential to consider that the accessibility of CT-guided PD can vary depending on the specific anatomical placement of the abscess and the patient’s habitus, which can affect the visibility and reach required for adequate drainage. Moreover, larger abscesses, typically more accessible to drain, may not accurately represent the success rates in more challenging cases with smaller or unfavorably located abscesses. We aim to address these complexities by emphasizing the need for a tailored approach in selecting drainage techniques, considering the specific clinical circumstances and limitations posed by patient anatomy and abscess characteristics.

In our analysis of percutaneous drainage modalities for psoas muscle abscesses, we recognize that outcomes are intricately linked to factors such as patient comorbidities, disease severity, the selection and susceptibility of antimicrobial agents, and the pathogen’s virulence. Although our data extraction aimed to include patient comorbidities and causative agents, the available data were insufficient for a definitive conclusion. However, asserting that healthier patients and larger abscesses generally exhibit better outcomes is reasonable. 

Our findings suggest that CT-guided techniques provide greater precision, although this is not to underestimate the efficacy of US-guided techniques. Instead, our goal is to enhance surgical precision across all techniques. The findings consistently indicate that imaging techniques, particularly MRI, enhance diagnostic accuracy and potentially improve outcomes in both US-guided and CT-guided drainage procedures. Notably, MRI diagnosis has shown to be advantageous across different modalities, suggesting its value in managing complex cases where anatomical challenges, abscess size, and the patient’s general health are significant factors.

Integrating an O-arm scan with MRI intraoperatively, combined with the navigation system, has already proven to be beneficial in managing various liver, kidney, and spinal column tumors, leveraging the distinct advantages of CT and MRI during surgical procedures [25,26]. Utilizing MRI’s detailed soft tissue visualization alongside the precise anatomical detail provided by CT ensures highly accurate targeting and the adequate drainage of abscesses. We believe that this multimodal approach can significantly advance the drainage of psoas muscle abscesses. The addition of a navigation system enhances procedural accuracy, minimizing the risk of complications and ensuring the optimal placement of drainage catheters. Furthermore, the capability to perform a post-drainage intraoperative Cone Beam CT (CBCT) scan allows for the immediate verification of the catheter’s placement and the effectiveness of the drainage, thereby ensuring comprehensive treatment. The successful application of CBCT-MRI fusion techniques in other complex surgical contexts underscores its potential to significantly improve outcomes in the drainage of challenging psoas muscle abscesses.

Furthermore, we advocate for the adoption of CBCT-MR fusion techniques with SS8 in anatomically challenging or smaller abscesses, which may offer enhanced precision in difficult cases by combining the strengths of both imaging modalities. This recommendation is intended not as a conclusive judgment but as a contribution to ongoing discussions and advancements in the management of challenging psoas abscesses. 

## 5. Conclusions

While our study design and the retrospective nature of the included studies limit the ability to assert the superiority of one intervention over another definitively, the consistent results across different studies advocate for a multimodal approach that leverages the strengths of both MRI for diagnostic accuracy and CT for effectively guiding drainage procedures. As we move forward, refining techniques for managing challenging abscesses in patients with poorer prognoses, often due to comorbidities, difficult abscess locations, or small abscess sizes, is essential. Consequently, we recommend integrating CBCT-MRI fusion techniques in drainage to enhance precision and improve outcomes in these complex cases.

## Figures and Tables

**Table 1:** An overview of the studies that involved CT-guided PD. {} the number of patients.

Authors	Reference	Patients	Mean Age (Years)	Male/Female	Location	Abscess Size (cm)	Volume Drained (mL)	Etiology	Comorbidities	Complications	Follow-Up Period (Months)
6 CT—MRI											
(Asai et al., 2013)	[27]	19	-	-	Iliopsoas	-	-	-	-	-	-
(Zou et al., 2017)	[35]	16	37.6	9 Males 7 Females	T12-L1 {3}, L1-L2 {4}, L2-L3 {3}, L3-L4 {2}, L4-L5 {4}; bilateral [10]		>100	Spinal tuberculosis	-	-	26.6
(Dietrich et al., 2013)	[29]	23	62	12 Males 11 Females	-		-	Spondylodiscitis, GI infections, and postoperative septic complications	-	-	-
(Dinç et al., 2002)	[22]	21	42.9	13 Males 8 Females	Right psoas muscle {5}, right iliopsoas muscle {6}, left psoas muscle {4}, left iliopsoas muscle {8}, paravertebral {3}, bilateral {5}		321	Spinal tuberculosis, spondylodiskitis	-	-	24
(Fesatidou et al., 2022)	[30]	8	52.6	4 Males 4 Females	Bilateral {2}	6.3	-	Spinal tuberculosis, spondylodiscitis	Immunodeficiency {6}	-	6
(Kinoshita et al., 2016)	[32]	7	62.8	6 Males 1 Female	Psoas muscle {7}, iliopsoas muscle {3}, bilateral {1}	5.1	-	-	-	-	-
1 MRI											
(Tofuku et al., 2014)	[33]	23	69	13 Males 10 Females	Right iliopsoas muscle {6}, left iliopsoas muscle {11}, bilateral {6}	4.8	-	Pyogenic spondylitis	Diabetes {7}, renal failure {3}		27.7
4 CT											
(Cantasdemir et al., 2003)	[21]	22	36	10 Males 11 Females	Iliac muscle {2}, psoas muscle {14}, iliopsoas muscle {6}, bilateral {1}	9.2	890	Tuberculous sacroiliitis (TB), acute pyelonephritis (S. Aureus)	Diabetes {1}, systemic lupus erythematosus {1}	Recurrence {3}, catheter complication {5}, death due to sepsis {1}	49.6
(Hayashi et al., 2022)	[31]	8	-	-	Lumbar level {1}, lumbar and pelvic level {7}, bilateral {1}	9	-	-	-	-	-
(Yacoub et al., 2008)	[34]	27	-	-	-	6	-	-	-	Recurrence {4}	-
(Baier et al., 2006)	[28]	8	-	-	-	-	-	-	-	Recurrence {4}	-

**Table 2:** An overview of the studies that involved US-guided PD. {} the number of patients.

Authors	Reference	Patients	Mean Age (Years)	Male/ Female	Location	Abscess Size (cm)	Volume Drained (mL)	Etiology	Comorbidities	Complications	Follow-Up Period (Months)
1 CT/MRI											
(Xu et al., 2022)	[36]	18	-	-	-	-	-	-	-	Recurrence {2}	-
1 MRI											
(Lai et al., 2018)	[37]	36	42.5	20 Males 16 Females	-	-	-	Spinal tuberculosis	-	-	**13**
4 US											
(Thakral et al., 2022)	[38]	15	-	-	-	-	-	-	-	Recurrence {2}, catheter complications {2}	-
(Aboobakar et al., 2018)	[12]	16	-	-	-	-	-	-	-	Recurrence {2}	-
(Kang et al., 1998)	[39]	14	6	6 Males 8 Females	Iliopsoas muscle {14}; bilateral {2}	-	55	-	-	Recurrence [3], death {1}	-
(Khedkar et al., 2018)	[40]	13	-	-	-		20–300	-	-	Recurrence {4}	-
1 UKNOWN											
(Shah et al., 2023)	[41]	25	-	-	-	-	-	-	-	Recurrence {5}, death {1}	-

**Table 3:** An overview of the studies that involved CT-guided combined with US-guided PD. [] {}the number of patients.

Authors	Reference	Patients	Mean Age (Years)	Male/Female	Location	Abscess Size (cm)	Volume Drained (mL)	Etiology	Comorbidities	Complications	Follow-Up Period (Months)
1 CT											
(Suzuki et al., 2015)	[42]	14	70	7 Males 7 Females	Bilateral {5}	-	-	Spondylodiscitis, GI perforation, UTI spreading, and postoperative abscess	Diabetes, thyroid cancer, ovarian cancer, hypertension, dyslipidemia, liver cirrhosis, spinal cord injury, hyperuricemia	Death {1}	-
1 CT/US											
(Dahniya et al., 1999)	[43]	6	43	2 Males 4 Females	Bilateral {1}	-	-	Tuberculosis	Diabetes, severe cardiac disease, and arteriopathy {1}	-	-

## Data Availability

All data of this meta-analysis are available from the authors upon reasonable request.

## References

[1] Bartolo D.C., Ebbs S.R., Cooper M.J. (1987). Psoas Abscess in Bristol: A 10-Year Review. Int. J. Color. Dis..

[2] Gruenwald I., Abrahamson J., Cohen O. (1992). Psoas Abscess: Case Report and Review of the Literature. J. Urol..

[3] Wong O.F., Ho P.L., Lam S.K. (2013). Retrospective Review of Clinical Presentations, Microbiology, and Outcomes of Patients with Psoas Abscess. Hong Kong Med. J..

[4] Xu B.Y., Vasanwala F.F., Low S.G. (2019). A Case Report of an Atypical Presentation of Pyogenic Iliopsoas Abscess. BMC Infect. Dis..

[5] Khatoon S., Ahmedani A., Unar S.K., Memon R.A., Khatti S. (2021). Sundesh Outcome of Percutaneous Ultrasound Guided Aspiration versus Open Surgical Drainage of Psoas Muscle Abscess. Ann. PIMS-Shaheed Zulfiqar Ali Bhutto Med. Univ..

[6] Lai Y.-C., Lin P.-C., Wang W.-S., Lai J.-I. (2011). An Update on Psoas Muscle Abscess: An 8-Year Experience and Review of Literature. Int. J. Gerontol..

[7] Taiwo B. (2001). Psoas Abscess: A Primer for the Internist. S. Med. J..

[8] Mallick I.H., Thoufeeq M.H., Rajendran T.P. (2004). Iliopsoas Abscesses. Postgrad. Med. J..

[9] Shields D., Robinson P., Crowley T.P. (2012). Iliopsoas Abscess—A Review and Update on the Literature. Int. J. Surg..

[10] Huang W., Wu T., Gao G., Chen W., Wu J., Cheng X. (2016). Psoas Abscess and Osteomyelitis of Femoral Head Due to Ileocecal Adenocarcinoma: A Case Report. Int. J. Clin. Exp. Med..

[11] Ricci M.A., Rose F.B., Meyer K.K. (1986). Pyogenic Psoas Abscess: Worldwide Variations in Etiology. World J. Surg..

[12] Aboobakar R., Cheddie S., Singh B. (2018). Surgical Management of Psoas Abscess in the Human Immunodeficiency Virus Era. Asian J. Surg..

[13] Kim Y.J., Yoon J.H., Kim S.I., Wie S.H., Kim Y.R. (2013). Etiology and Outcome of Iliopsoas Muscle Abscess in Korea; Changes over a Decade. Int. J. Surg..

[14] Pombo F., Martín-Egaña R., Cela A., Díaz J.L., Linares-Mondéjar P., Freire M. (1993). Percutaneous Catheter Drainage of Tuberculous Psoas Abscesses. Acta Radiol..

[15] Tabrizian P., Nguyen S.Q., Greenstein A., Rajhbeharrysingh U., Divino C.M. (2009). Management and Treatment of Iliopsoas Abscess. Arch. Surg..

[16] Zissin R., Gayer G., Kots E., Werner M., Shapiro-Feinberg M., Hertz M. (2001). Iliopsoas Abscess: A Report of 24 Patients Diagnosed by CT. Abdom. Imaging.

[17] Qureshi N.H., O’Brien D.P., Allcutt D.A. (2000). Psoas Abscess Secondary to Discitis: A Case Report of Conservative Management. J. Spinal Disord..

[18] Wu T.L., Huang C.H., Hwang D.Y., Lai J.H., Su R.Y. (1998). Primary Pyogenic Abscess of the Psoas Muscle. Int. Orthop..

[19] Huang J.J., Ruaan M.K., Lan R.R., Wang M.C. (2000). Acute Pyogenic Iliopsoas Abscess in Taiwan: Clinical Features, Diagnosis, Treatments and Outcome. J. Infect..

[20] Procaccino J.A., Lavery I.C., Fazio V.W., Oakley J.R. (1991). Psoas Abscess: Difficulties Encountered. Dis. Colon. Rectum.

[21] Cantasdemir M., Kara B., Cebi D., Selcuk N.D., Numan F. (2003). Computed Tomography-Guided Percutaneous Catheter Drainage of Primary and Secondary Iliopsoas Abscesses. Clin. Radiol..

[22] Dinç H., Ahmetoğlu A., Baykal S., Sari A., Sayil O., Gümele H.R. (2002). Image-Guided Percutaneous Drainage of Tuberculous Iliopsoas and Spondylodiskitic Abscesses: Midterm Results. Radiology.

[23] Gervais D.A., Hahn P.F., O’Neill M.J., Mueller P.R. (2002). Percutaneous Abscess Drainage in Crohn Disease: Technical Success and Short- and Long-Term Outcomes during 14 Years. Radiology.

[24] Haaga J.R. (1990). Imaging Intraabdominal Abscesses and Nonoperative Drainage Procedures. World J. Surg..

[25] Al-Smadi M.W., Kozma I., Aslan S., Bölöni B., Viola Á. (2023). Percutaneous Superimposed O-Arm-MRI-Navigated Biopsy for Spinal Column Pathologies. Diagnostics.

[26] Aslan S., Al-Smadi M.W., Kozma I., Viola Á. (2023). Enhanced Precision and Safety in Thermal Ablation: O-Arm Cone Beam Computed Tomography with Magnetic Resonance Imaging Fusion for Spinal Column Tumor Targeting. Cancers.

[27] Asai N., Ohkuni Y., Yamazaki I., Kawamura Y., Kaneko N., Aoshima M. (2013). Clinical Manifestations and Prognostic Factor of Iliopsoas Abscess. J. Glob. Infect. Dis..

[28] Baier P.K., Arampatzis G., Imdahl A., Hopt U.T. (2006). The Iliopsoas Abscess: Aetiology, Therapy, and Outcome. Langenbecks Arch. Surg..

[29] Dietrich A., Vaccarezza H., Vaccaro C.A. (2013). Iliopsoas Abscess: Presentation, Management, and Outcomes. Surg. Laparosc. Endosc. Percutan. Tech..

[30] Fesatidou V., Petsatodis E., Kitridis D., Givissis P., Samoladas E. (2022). Minimally Invasive Outpatient Management of Iliopsoas Muscle Abscess in Complicated Spondylodiscitis. World J. Orthop..

[31] Hayashi N., Takeuchi Y., Morishita H., Ehara N., Yamada K. (2022). CT-Guided Femoral Approach for Psoas Muscle Abscess Drainage. Cardiovasc. Interv. Radiol..

[32] Kinoshita M., Takao S., Takechi K., Takeda Y., Miyamoto K., Yamanaka M., Akagawa Y., Iwamoto S., Osaki K., Tani H. (2016). Percutaneous Drainage of Psoas and Iliopsoas Muscle Abscesses with a One-Step Technique under Real-Time Computed Tomography Fluoroscopic Guidance. J. Med. Investig..

[33] Tofuku K., Koga H., Komiya S. (2014). Percutaneous Drainage Combined with Hyperbaric Oxygen Therapy for Pyogenic Spondylitis with Iliopsoas Abscess. Asian Spine J..

[34] Yacoub W.N., Sohn H.J., Chan S., Petrosyan M., Vermaire H.M., Kelso R.L., Towfigh S., Mason R.J. (2008). Psoas Abscess Rarely Requires Surgical Intervention. Am. J. Surg..

[35] Zou D.X., Zhou J.L., Zhou X.X., Jiang X.B. (2017). Clinical Efficacy of CT-Guided Percutaneous Huge Ilio-Psoas Abscesses Drainage Combined with Posterior Approach Surgery for the Management of Dorsal and Lumbar Spinal Tuberculosis in Adults. Orthop. Traumatol. Surg. Res..

[36] Xu C., Wang S., Wu W., Hui T., Ren W., Yang X., Chen H., Zheng W., Yin Q., Pan H. (2022). Aetiology and Clinical Characteristics of Psoas Abscesses in China, 2011–2021: A Retrospective Study. Res. Sq..

[37] Lai Z., Shi S., Fei J., Han G., Hu S. (2018). A Comparative Study to Evaluate the Feasibility of Preoperative Percutaneous Catheter Drainage for the Treatment of Lumbar Spinal Tuberculosis with Psoas Abscess. J. Orthop. Surg. Res..

[38] Thakral A., Prasad D., Katyal S., Kumar A. (2022). Characteristics and Outcomes of Psoas Abscess: Experience from a Tertiary Care Center in North India. Cureus.

[39] Kang M., Gupta S., Gulati M., Suri S. (1998). Ilio-Psoas Abscess in the Paediatric Population: Treatment by US-Guided Percutaneous Drainage. Pediatr. Radiol..

[40] Khedkar K., Sharma C., Kumbhar V., Waghmare M., Dwivedi P., Gandhi S., Shah H. (2018). Management of Paediatric Psoas Abscess: Our Experience. J. Pediatr. Neonatal Individ. Med. (JPNIM).

[41] Shah A.G., Prajapati P., Kakadiya H. (2023). Comparative Study of Surgical Drainage versus Percutaneous Drainage of Psoas Abscess. Natl. J. Physiol. Pharm. Pharmacol..

[42] Suzuki K., Yamaguchi T., Iwashita Y., Yokoyama K., Fujioka M., Katayama N., Imai H. (2015). Case Series of Iliopsoas Abscesses Treated at a University Hospital in Japan: Epidemiology, Clinical Manifestations, Diagnosis and Treatment. Intern. Med..

[43] Dahniya M.H., Hanna R.M., Grexa E., Cherian M.J., Niazy M.N., Badr S., Ibrahim F., al-Othman A.N. (1999). Percutaneous Drainage of Tuberculous Iliopsoas Abscesses under Image Guidance. Australas. Radiol..

[44] Sato T., Kudo D., Kushimoto S. (2021). Epidemiological Features and Outcomes of Patients with Psoas Abscess: A Retrospective Cohort Study. Ann. Med. Surg..

[45] Martins D.L.N., de Assis Cavalcante F., Falsarella P.M., Rahal Junior A., Garcia R.G. (2018). Percutaneous drainage of iliopsoas abscess: An effective option in cases not suitable for surgery. Einstein.

[46] Benkhadoura M.O., El-Mogassabi A.H., Mansor S.M., Abuzaid I.A., Manita M.A., Etabbal A.M., Elgazwi K.K., Elshaikhy A.I. (2019). Iliopsoas Abscess: Clinical Presentation, Management, and Outcome. Int. Surg. J..

[47] Paley M., Sidhu P.S., Evans R.A., Karani J.B. (1997). Retroperitoneal Collections—Aetiology and Radiological Implications. Clin. Radiol..

[48] Jiang K., Zhang W., Fu G., Cui G., Li X., Ren S., Fu T., Geng L. (2022). Ultrasound-Guided Percutaneous Drainage of Iliopsoas Abscess With Septicemia in an Adolescent: A Case Report and Literature Review. Front. Surg..

[49] Bresee J.S., Edwards M.S. (1990). Psoas Abscess in Children. Pediatr. Infect. Dis. J..

[50] Rodrigues J., Iyyadurai R., Sathyendra S., Jagannati M., Prabhakar Abhilash K.P., Rajan S.J. (2017). Clinical Presentation, Etiology, Management, and Outcomes of Iliopsoas Abscess from a Tertiary Care Center in South India. J. Fam. Med. Prim. Care.

[51] Pérez-López L.M., Vara-Patudo I., Torner-Rubies F., Moreno-Romo D., Cabo L.S., Fortuny C., Knörr G. (2017). Pediatric Psoas Abscess, Early Diagnosis of a Challenging Condition. J. Acute Med..

[52] Duani H., Nunes V.R.T., Assumpção A.B., Saraiva I.S.B., Rosa R.M., Neiva A.M., Pedroso E.R.P. (2012). Bilateral Paracoccidioidomycotic Iliopsoas Abscess Associated with Ileo-Colonic Lesion. Rev. Soc. Bras. Med. Trop..

[53] Sartelli M. (2010). A Focus on Intra-Abdominal Infections. World J. Emerg. Surg..

[54] De Filippo M., Puglisi S., D’Amuri F., Gentili F., Paladini I., Carrafiello G., Maestroni U., Del Rio P., Ziglioli F., Pagnini F. (2021). CT-Guided Percutaneous Drainage of Abdominopelvic Collections: A Pictorial Essay. Radiol. Med..

[55] Bickle I. CT Guided Percutaneous Drainage|Radiology Reference Article|Radiopaedia.Org. https://radiopaedia.org/articles/ct-guided-percutaneous-drainage?lang=us.

[56] Chern C.-H., Hu S.-C., Kao W.-F., Tsai J., Yen D., Lee C.-H. (1997). Psoas Abscess: Making an Early Diagnosis in the ED. Am. J. Emerg. Med..

[57] Stern J.J., Stoopack P.M. (1988). Magnetic Resonance Imaging in the Diagnosis of a Psoas Abscess. J. Comput. Tomogr..

[58] Akhaddar A., Hall W., Ramraoui M., Nabil M., Elkhader A. (2018). Primary Tuberculous Psoas Abscess as a Postpartum Complication: Case Report and Literature Review. Surg. Neurol. Int..

